# Differential coexpression in human tissues and the confounding effect of mean expression levels

**DOI:** 10.1093/bioinformatics/bty538

**Published:** 2018-07-03

**Authors:** Marjan Farahbod, Paul Pavlidis

**Affiliations:** 1 Graduate program in Bioinformatics, University of British Columbia, Vancouver, Canada; 2 Department of Psychiatry, University of British Columbia, Vancouver, Canada; 3 Michael Smith Laboratories, University of British Columbia, Vancouver, Canada

## Abstract

**Motivation:**

Differential coexpression—the alteration of gene coexpression patterns observed in different biological conditions—has been proposed to be a mechanism for revealing rewiring of transcription regulatory networks. Despite wide use of methods for differential coexpression analysis, the phenomenon has not been well-studied. In particular, in many applications, differential coexpression is confounded with differential expression, that is, changes in average levels of expression across conditions. This confounding, despite affecting the interpretation of the differential coexpression, has rarely been studied.

**Results:**

We constructed high-quality coexpression networks for five human tissues and identified coexpression links (gene pairs) that were specific to each tissue. Between 3 and 32% of coexpression links were tissue-specific (differentially coexpressed) and this specificity is reproducible in an external dataset. However, we show that up to 75% of the observed differential coexpression is substantially explained by average expression levels of the genes. ‘Pure’ differential coexpression independent from differential expression is a minority and is less reproducible in external datasets. We also investigated the functional relevance of pure differential coexpression. Our conclusion is that to a large extent, differential coexpression is more parsimoniously explained by changes in average expression levels and pure links have little impact on network-based functional analysis.

**Supplementary information:**

Supplementary data are available at *Bioinformatics* online.

## 1 Introduction

Coexpression networks have been adopted as a convenient representation of the pairwise similarities between gene RNA levels in transcriptomic datasets (Gillis and Pavlidis, 2011; Lee *et al.*, 2004). A key feature of coexpression is that pairs of coexpressed genes have a tendency to be functionally related (Brown *et al.*, 2000; Eisen *et al.*, 1998; Wen *et al.*, 1998). For this reason coexpression networks have been widely used in computational function prediction frameworks, based on the Guilt By Association (GBA) principle (Mostafavi *et al.*, 2008; Pavlidis and Gillis, 2013; Quackenbush, 2003) and various types of coexpression analysis are prominent features of many transcriptomic studies (Amar *et al.*, 2013; de la Fuente, 2010; Gillis and Pavlidis, 2009; Langfelder and Horvath, 2008).

**Fig. 1.**
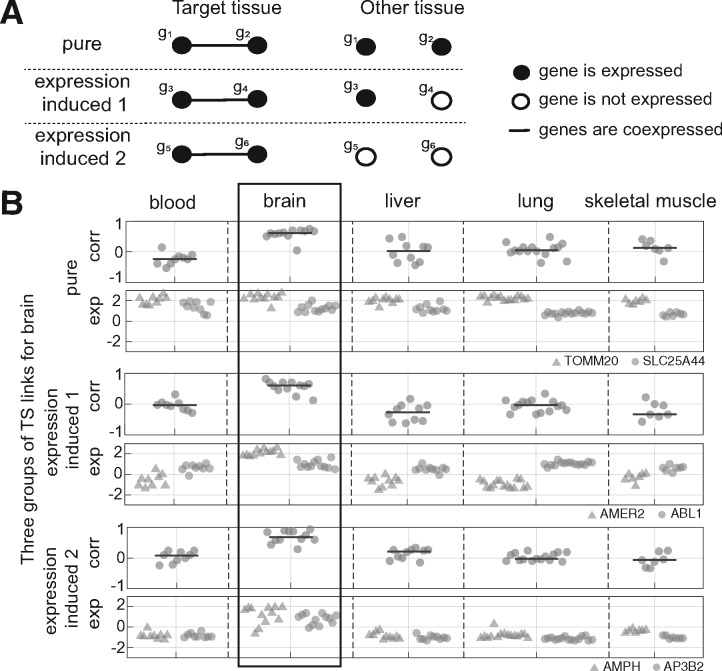
Different types of tissue-specific links. (**A**) Schematic representation of the effect of the average expression levels on differential coexpression, in three extreme cases. In reality links lie on a continuum among these classes. (**B**) Examples of the link classes from our data, with brain being the target tissue. Corr: correlation. Exp: expression levels. Gene symbols are indicated in the legend at right. Top panel: ‘pure’ differential coexpression between TOMM20 and SLC25A44. The two genes have moderate to high expression levels in all the tissues, but correlation values in brain are higher. Middle panel: expression-induced 1: AMER2 is only expressed in brain. Bottom panel: expression-induced 2: both AMPH and AP3B2 are only expressed in brain

An extension of coexpression analysis is differential coexpression analysis (DCA), referring to changes in coexpression between different conditions. DCA is commonly used as a complementary tool to differential expression analysis (DEA), where it is applied in an effort to reveal changes in regulatory ‘wiring’ between genes which is not otherwise captured through DEA (Amar *et al.*, 2013; de la Fuente, 2010; Gaiteri *et al.*, 2014; Gillis and Pavlidis, 2009; Kaushik *et al.*, 2015; Kostka and Spang, 2004; Mentzen *et al.*, 2009). An issue central to this paper is that differential coexpression and differential expression are often confounded. At one extreme, two genes cannot be coexpressed if one (or both) of the genes is not expressed at all and some observed differential coexpression is simply explained by the absence of expression (see Fig. 1, ‘expression-induced’ links). Also in general the variance of expression is frequently a function of expression level (Law *et al.*, 2014; Rocke and Durbin, 2001). Examining differential coexpression in the absence of differential expression is also appealing for reasons of parsimony: if two genes are found to be differentially expressed, it is not necessarily clear what new information is gained if one (or both) of them are also differentially coexpressed. For these reasons, it is of interest to investigate the degree to which differential coexpression is explained by differential expression, and to isolate cases where differential coexpression is not accompanied by changes in mean expression level. The latter ‘pure differential coexpression’ (Fig. 1) would be good candidates for instances of ‘rewiring’, though even then the interpretation is not necessarily straightforward.

**Fig. 2.**
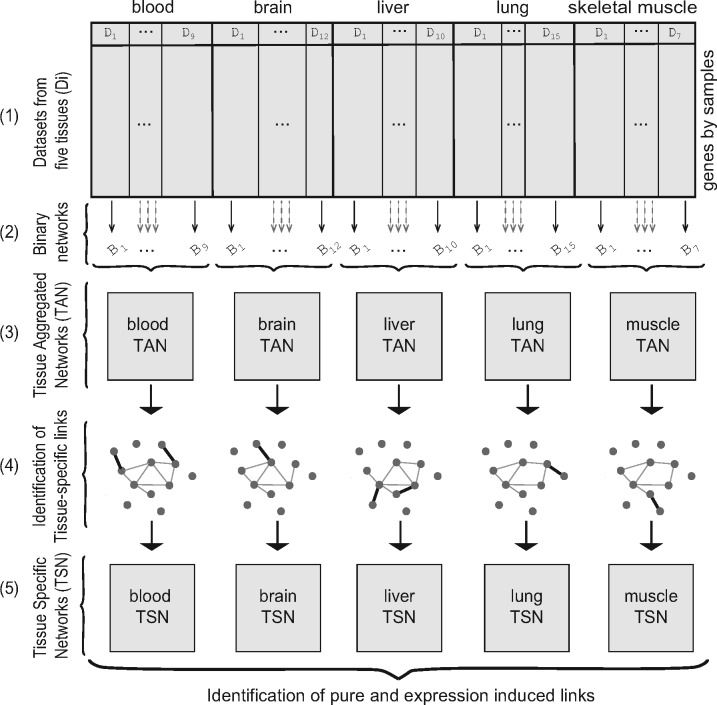
Overview of the network construction approach. Tissue aggregated networks (TANs) are built by combining binary networks from individual datasets. In each TAN, tissue-specific links are identified based on their correlation values in all the datasets. The tissue-specific links comprise tissue specific networks (TSNs)

In this paper, we study differential coexpression in different human tissues with a specific goal of accounting for the impact of changes in mean expression levels, and to thereby identify ‘pure’ differential coexpression between tissues. Tissue based coexpression networks have been previously used to improve GBA-based prediction for tissue related disease and functions (Greene *et al.*, 2015; Guan *et al.*, 2012; Pierson *et al.*, 2015). They have also been used in integrated or individual frameworks for studying tissue-specific gene regulation (Saha *et al.*, 2017; Sonawane *et al.*, 2017). We hypothesized that different tissues should be a relatively rich source of cases of ‘network rewiring’, due to their wide biological differences, and establishment of the principles would open the door to application in other scenarios such as different disease conditions. To this end, we built coexpression networks for five human tissues by combining coexpression networks from multiple datasets [tissue aggregated networks (TANs)]. We identified tissue-specific links in the TANs, forming tissue-specific networks (TSNs; Fig. 2). We then modeled how much of the observed differential coexpression between tissues (tissue-specific links) is predictable by the mean expression level of the genes. Based on this, we identified pure links as a subset of TSN links for each tissue. We found that the average expression level of genes is indeed a strong confound for much tissue-specific coexpression and that pure links are rare. We show that our TAN, TSN and pure links are generally reproducible in external datasets, while pure links are reproduced to a lesser extent. We further searched for the biological significance of the pure links and compared them to the expression-induced links regarding their functional implications in TSNs.

## 2 Materials and methods

All code and key data files for the analyses presented are available as Supplementary Materials.

### 2.1 Datasets

We used two collections of expression datasets to build and examine the reproducibility of our networks, a collection of Affymetrix microarray datasets and RNA-seq data from GTEx. The TANs and TSNs were built from Affymetrix microarray datasets. The pure links are also identified based on the Affymetrix datasets and GTEx data was used for validation of these three groups of links. The Affymetrix datasets consist of 53 datasets that used Affymetrix U133 Plus 2.0 GeneChips, reanalyzed from raw data (see Supplementary Tables S1–S5, Supplementary Fig. S1, Supplementary Section 1). The final data matrix included 18 494 genes and 3563 samples. For each tissue, genes were filtered based on their expression level and only genes which were marked as expressed were considered for building the coexpression networks. For GTEx data, we used the gene-level expression data from version 6, downloaded from the GTEx portal (Lonsdale *et al.*, 2013) for the five tissues blood, brain-cortex, liver, lung and skeletal-muscle.

### 2.2 Tissue aggregated networks (TAN)

**Fig. 3.**
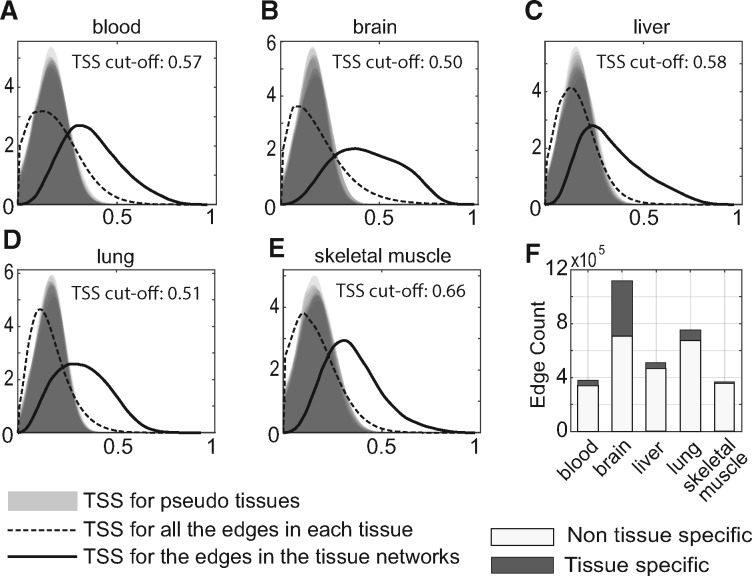
Tissue specificity score (TSS) of links. (**A–E**) For each tissue, the distribution of tissue specificity scores is plotted for all the links in its TAN (solid line) as compared to 10 generated nulls (pseudo-tissues; gray shading) and all the expressed gene pairs in the tissue (dashed line). (**F**) Summary of links considered tissue-specific or not, selected at FDR.01 compared to the null

The process of network generation is outlined in Figure 2. TANs were built by aggregating binary coexpression networks from Affymetrix datasets for each tissue (Fig. 2–1), similar to the approach used in Lee *et al.* (2004). Specifically, from each dataset, a raw coexpression matrix was first computed using pairwise Pearson correlations between the gene pairs. Binary networks were built from these raw networks by selecting the gene pairs having coexpression value >90th percentile within the dataset (Fig. 2–2). These selected pairs are referred throughout as ‘links’. For each tissue those links present in at least in *n* binary networks were marked as present. The value of *n* was chosen for each tissue to maintain a false discovery rate (FDR) of 10^−4^, using the binomial distribution as the null (Fig. 2–3). This FDR was chosen to obtain networks of reasonable density, but our key findings are not affected by the exact choice.

### 2.3 Identification of tissue-specific links

Tissue-specific links were identified for each tissue as a subset of the links in the tissue’s TAN. This process has two steps: (i) measuring a tissue-specificity score (TSS) for each link and (ii) setting thresholds for TSSs to control false discovery rates.

#### 2.3.1 Measuring the TSS for each of the links in TANs

Our goal was to capture pairs of genes which consistently have relatively high coexpression in one tissue (the *target* tissue), while having consistently lower coexpression in all the *other* tissues. To implement this, we first define the correlations for a pair of genes *(g_i_, g_j_)* in each of the *k* data sets, referred to as *S_ij_*:

(1) $$S_{ij}=\left\{corr\left(i,j,k\right)\;\right|\;i\neq j,\;1\;\leq k\leq 53\}.$$

Where *corr(i, j, k)* is the Pearson correlation value between the genes *g_i_* and *g_j_* from dataset *k* (*D*_k_). We modify these raw correlation values for each of the links in two steps. The first step is the normalization between the datasets using a binned rank transformation. The *corr(i, j, k)* is replaced with the per mille it belongs to between coexpression values from all the expressed genes in *D*_k_ (this binning was used to simplify the implementation). Since we were attempting to capture the presence of coexpression in the target tissue and the absence of it in the other tissues, negative correlations were not of interest, and they could affect the measurements as outliers. Therefore, all negative correlations were assigned to bin 500, corresponding to approximately a zero correlation. More formally:

(2) $${SB}_{ij}=\left\{\max\left(bin\left(r,k\right),\;500\right)\;\right|\;r\;\in \;S_{ij},\;i\neq j,\;1\;\leq k\leq 53\}.$$

Where *bin(r, k)* gives the per mille of the correlation value *r* in dataset *k*. Next, having the tissue *t*_m_ and the link *l_ij_*, we define two subsets of *SB_i, j_* as follows:

(3) $${CT}^{t}=\left\{r_{k}\;\right|\;r_{k}\;\in \;{SB}_{ij},\;dataset\;k\;is\;from\;tissue\;t\}$$

(4) $$\bar{{CT}^{t}}=\;{SB}_{ij}-{CT}^{t}.$$

From this, the TSS of a given pair of genes *(g_i_, g_j_)* and a given tissue *t*_m_ is calculated as follows:

(5) $$TSS\left(g_{i},g_{j},t_{m}\right)=\;\frac{\sum_{p\in {CT}^{t}}\sum_{q\in \bar{{CT}^{t}}}f(r_{p}-r_{q})}{\left|{CT}^{t}\right|\left|\bar{{CT}^{t}}\right|}$$

(6) fx= x, x>00, x≤0.

We also applied an alternative method using the *P*-value from a Wilcoxon rank sum test, comparing the ranks of the correlations for the gene pair in one tissue to that in all others. The results of the two approaches are highly correlated (Supplementary Fig. S3) and we focused on the TSS as defined above.

#### 2.3.2 Controlling false discovery rates for TSSs

To assess the null distribution of TSS, we created random subsets of the 53 datasets pseudo-tissues and computed TSSs for each of them the same way as the real tissues. Each pseudo-tissue was constructed to have all five tissues represented equally. We built 30 sets of pseudo-tissues for each tissue, with the same count of datasets as the tissue. The TSS-FDR for the tissue *t* at a given value *tss* is calculated as follows:

(7) $${FDR}_{t}\left(tss\right)=\;\left(\sum_{pt=1}^{30}\left|{ϒ}_{pt}^{tss}\right|/30\right)\frac{1}{\left|{ϒ}_{t}^{tss}\right|}.$$

Where ${ϒ}_{pt}^{tss}$ is the set of TSS values for pseudo-tissue *pt* which are greater than *tss*. Similarly, ${ϒ}_{t}^{tss}$ is defined for tissue *t*. We selected our TSS threshold for the FDR 0.01 for each tissue. Selected TS links were further filtered to select those with their TSS < 0.4 in all the other tissues.

### 2.4 Modeling the effect of expression level on tissue-specific links

We used a linear model to examine the predictive power of expression level of pairs of genes for their coexpression value:

(8) $$bin\left(corr\left(i,j\right),k\right)=\beta_{0}+\beta_{1}\left(e_{ik}+e_{jk}\right){+\beta }_{2}\left|e_{ik}-e_{jk}\right|,\;i\neq j,\;1\leq k\leq 53.$$

Where *e*_ik_ is the mean expression level of gene *i* in dataset *k*. This model captures the predictive value of the total expression of the two genes (*ß*_1_) and their difference (*ß*_2_). The model was fit to all the links in the five TSNs. The *R*^2^ of the model fit is a continuous measurement of the predictability of each of link based on the expressed level of their genes. These *R^2^* values were compared to a null *R^2^*, which was computed by shuffling the mean expression level of the genes across datasets and refitting the models.

### 2.5 Coexpression networks built from GTEx datasets for validation

We built binary and raw coexpression networks for the five GTEx datasets. Raw coexpression matrices were computed with the Pearson correlation of the gene expression profiles. To create binary networks, the threshold was selected so that each resulting network had the same density to the TAN of the tissue it was compared to.

## 3 Results

### 3.1 Tissue aggregated networks (TANs)

Our primary analysis is based on 53 high-quality datasets representing five human tissues (brain, lung, skeletal-muscle, blood and liver), collected on Affymetrix GeneChips (7–15 datasets per tissue, 3563 samples in total, assaying 18 494 genes; Supplementary Fig. S1A). For each tissue, we identified ‘expressed’ genes based on their mean expression levels (Supplementary Table S6, Supplementary Material 1). We then built TANs for each tissue by aggregating binary coexpression networks constructed from each dataset for that tissue, retaining links that were observed in multiple data sets, where the threshold minimum number of dataset networks was set to control the false discovery rate (FDR < 10^−4^, Fig. 2, Methods 2.2). Each TAN has a different number of links (Supplementary Table S6), but this was not significantly correlated with the number of genes present in the networks or the count of datasets for each tissue. The TANs form the basis for the ensuing analysis.

### 3.2 Many links are tissue-specific

We next identified tissue-specific links in each TAN. Our definition of a tissue-specific link is a link which has higher correlation values in one tissue (the ‘target’ tissue) versus all the ‘other’ tissues, while allowing for a certain level of noise. To do so we computed a TSS for each TAN link and a tissue, based on the average of the positive difference between normalized correlation values from the target tissue versus the rest of the tissues (Methods 2.3). The TSS for a link and a tissue reflects its specificity to that tissue. We generated null distributions for TSS to control the FDR for identification of tissue-specific links (Methods 2.3). Figure 3A–E shows the distribution of TSS for pseudo-tissues, all the expressed gene pairs and the TAN links. We identified a fraction between 0.03 and 0.32 of the TAN links as tissue-specific (Supplementary Table S7, FDR < 0.01).

### 3.3 Expression level changes explains much of the tissue-specific coexpression

**Fig. 4.**
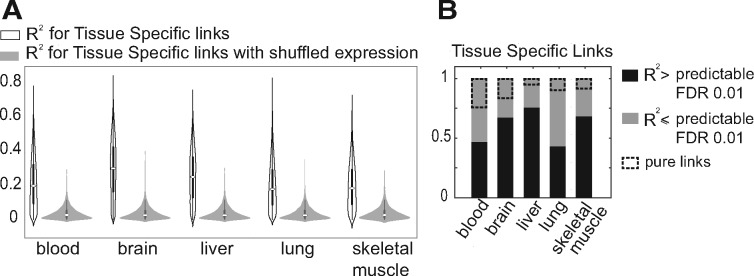
Identification of pure links. (**A**) Distribution of *R*^2^ for the TSN links in different tissues versus the null (links with shuffled expression). (**B**) Distribution of predictable and unpredictable TS links

To capture the relation between mean expression level and coexpression, we considered linear models in which the correlation between two genes is modeled as a function of the mean expression levels of the linked genes (Methods 2.4). The fitted models were then examined for how well they could explain the correlation of the link in the five tissues compared to a null using shuffled expression values. We find that for most of the tissue-specific links, the model *R*^2^ is higher compared to the null at FDR > 0.01 (Fig. 4A). This confirms that differences in mean expression can at least partly explain differences in coexpression.

Tissue-specific links that were not substantially associated with mean expression levels (having a low *R*^2^ for the model fit) are candidates for pure rewiring, since they are relatively uninfluenced by the confound of overall expression level shifts (as captured by the model). However, failure for the models to predict coexpression is not a sufficient constraint, so we also required that pure links have both genes marked as ‘expressed’ in all the tissues. This results in a smaller number of tissue-specific links, noted with dashed boxes in Figure 4B. Thus ‘pure differential coexpression’ links are by definition between genes expressed in all tissues, and variability in mean expression level across tissues does not appear to be associated with tissue-specificity of coexpression. We stress that this definition is fairly stringent, but still allows for some variation in mean expression levels. An example of a pure differentially-coexpressed link is shown in Figure 1 and additional examples are given in Supplementary Figure S6. We conclude that while much of the difference in coexpression among tissues is attributable to changes in mean expression level, there is a small number of links that appear to undergoing ‘pure’ differential coexpression.

### 3.4 Reproducibility and validation of the networks

**Fig. 5.**
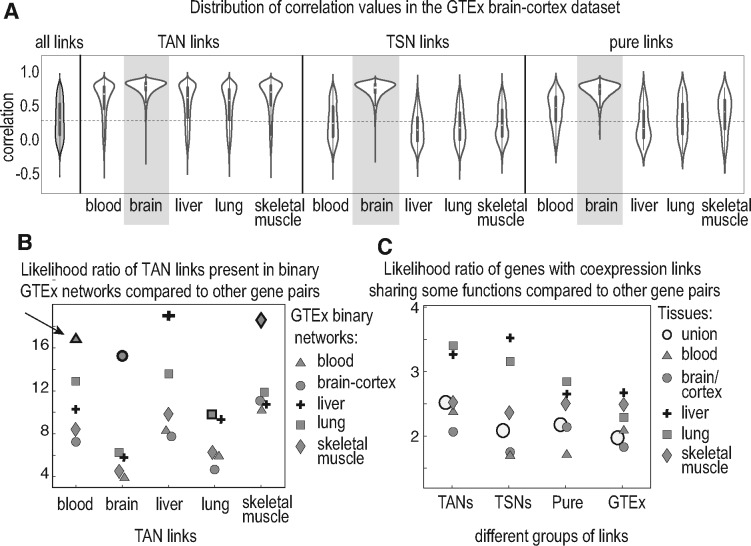
Reproducibility and functional relevance of our identified coexpression links. (**A**) Distribution of correlation values for TANs, TSNs and pure links in GTEx brain-cortex dataset. The filled gray violin is the distribution of all the correlation values in GTEx brain-cortex, included as the null reference. Distributions for the brain groups (highlighted) are denser at higher values compared to other tissues. Generally, links from all the five TANs have higher correlation values compared to the null. For all the TSNs the distribution is close to that from the null, except for the brain TSN. The pure TSN links show similar results to TSN links, although a bit weaker. (**B**) Summarization of the TAN reproducibility in GTEx binary networks. Shapes with highlighted borders represent links from the same tissue as the TAN. Arrow points to an example: the count of links from blood TAN in the binary GTEx blood network are >16 times more than expected by chance. (**C**) Likelihood ratio of having at least one same functional annotation between the genes that are connected in each of the networks versus the genes that are not connected. The blank circle represents the union of the networks

We examined the reproducibility of our TAN, TSN and pure networks in the five GTEx datasets: blood, brain-cortex, liver, lung and skeletal-muscle. We expected that links coming from a tissue will have generally higher correlation values in the same tissue in GTEx, compared to the other tissues. We examined the distribution of the correlation values in each of these GTEx datasets for any given groups of links (TAN, TSN and pure) from the five tissues. Figure 5A shows the distribution of correlation values for different groups of links in GTEx brain-cortex dataset. As expected, TAN, TSN and pure links from brain have generally much higher correlation values than the null in GTEx brain-cortex. TAN links from other tissues have higher correlation values than the null as well (though much less than the brain TAN), and this is expected due to the common links between TANs. On the contrary, TSN and pure links from other tissues have generally much lower correlation values than the null. This is also expected since the TSN and pure links from other tissues are not expected to have high correlation values in GTEx brain-cortex. Supplementary Figure S4 has similar plot for the rest of the tissues and Figure 5B summarizes the reproducibility of the TAN links in the five GTEx tissues in terms of likelihood ratio. On average, reproducibility of the pure links is less than TSN links (52% of tissue-specific links versus 25% of pure links are reproduced in GTEx - Supplementary Section 4).

One of the known characteristics of coexpression links is that they can capture functional similarity between genes. In a validation, we examined this for our binary networks and show that in the TANs, TSNs and GTEx binary networks, genes sharing a coexpression link are more likely to share some functional terms with each other than that expected by chance. This is also true for genes with pure links (Fig. 5C).

### 3.5 Characterization of pure differential coexpression

Despite the relatively small number of pure links, it is naturally of interest to understand if they have any particular biological relevance, but this presents a challenge. By definition, the genes participating in pure links are expressed in all of the tissues, and therefore tend not to have any easily identifiable tissue-specific function. Furthermore, 86% of genes expressed in all tissues (those eligible to have pure links) have at least one pure link (6658/7745) and 4267 have pure links in more than one tissue. We were unable to identify any distinguishing functional feature of the genes with pure links compared to the minority that lack them. They also do not stand out in terms of functional similarity: genes associated by a pure link are more likely to be functionally similar, but no more than the other classes of links (Fig. 5C). We also found that the neighborhoods of genes paired with pure links show higher topological overlap in the target tissue compared to the other tissues (Supplementary Fig. S5, Supplementary Material 5). This helps validate pure links as robust differential coexpression, but does not address their biological meaning.

We next turned to analyzing how pure links contribute to the network-level representation of gene function. We defined the enrichment of a term in a network-based on the number of links connecting the genes annotated with that term compared to a null, so a function is enriched if genes with that function have a statistically significant number of links joining them (Supplementary Section 6). We hypothesized that pure links in a tissue could contribute to tissue-specific enrichment of common cellular processes and tested this by examining what happens if we remove pure links from the network. We first found that 146, 91 and 43 functions are enriched exclusively in the brain, liver and lung TSNs, respectively (FDR < 0.01; too few functions were enriched for blood and skeletal-muscle for further analysis). When pure links were removed from these TSNs, the count of functions enriched in brain dropped by 20, by 3 in liver and by 4 in lung. The number of such ‘drop-out’ functions for brain and lung is less than those affected by removing equivalent numbers of random links (*P* < 0.05, Supplementary Section 6). It is also less than removing random sets of expression-induced links (*P* < 0.01, Supplementary Section 7). Enrichment of five functions in brain were sensitive only to the removal of pure links including ‘regulation of mitochondrion organization’ (GO: 0010821) and ‘phospholipid biosynthetic process’ (GO: 0008654—see Supplementary Tables; none of the enriched functions in lung and liver showed such sensitivity). We also performed an analysis of disease associations (rather than functions defined by the gene ontology; Supplementary Section 8). Brain and lung had 18 and 30 diseases exclusively enriched in their TSN. Of these, 1 and 5 (respectively) were sensitive to the removal of pure links. However, all of these terms were also sensitive to the removal of random sets of links and expression-induced links.

## 4 Discussion

In this paper, we studied coexpression in five human tissues in an attempt to document the effect of the mean expression level on tissue-specific coexpression. In each of the five tissues that we have studied, we were able to identify robust coexpression links which are, to great extent, reproducible in external datasets (TANs) and represent functional similarity between the genes. We were also able to identify tissue-specific links (TSNs). However, we found that, as hypothesized, the majority of differential coexpression between the tissues (tissue-specific links) can be substantially explained by changes in mean expression level of one or both genes and that cases of pure differential coexpression are relatively rare. Many of our pure links are reproduced in GTEx datasets but as a group, pure links contribute to the tissue-specific enrichment of a few functional terms while in comparison, expression-induced links contribute to the tissue-specific enrichment of many functional terms. Our work has implications for other studies of differential coexpression, as we now discuss.

We have stressed the importance of controlling for the confounding effects of mean expression level on coexpression. Some previous literature also distinguishes differential expression and differential coexpression and refer to their complementary role (Amar *et al.*, 2013; Gaiteri and Sibille, 2011; Lai *et al.*, 2004). The confounding role of the differential expression has also been noted in the tissue based coexpression networks, where It was observed that the coexpression modules in tissues are enriched with tissue-specific genes (Langfelder *et al.*, 2011; Pierson *et al.*, 2015). Despite this, we are aware of only one attempt to control for expression levels in DCA, in an analysis of a brain development data set (Crow *et al.*, 2016). More commonly differential expression is merely reported or commented upon alongside differential coexpression results (Hu *et al.*, 2009; Jiang *et al.*, 2016; Kostka and Spang, 2004; Mentzen *et al.*, 2009). But most studies of differential coexpression do not even go that far. We suggest that when differential expression is present, differential coexpression should be interpreted very carefully. This seems especially important given the biological relevance or meaning of differential coexpression has rarely matched the hopes of revealing ‘rewiring’. In fact, we are not aware of any case where ‘regulatory rewiring’ has been uncovered in an unbiased DCA, especially in the absence of differential expression.

One of the challenges in identifying differential coexpression is controlling sensitivity and specificity. To help control multiple test penalties that would be incurred by testing every pair of genes, most studies use data reduction approaches such as considering only a subset of genes, or ‘gene modules’ in the networks, comparing their attributes or their preservation (Langfelder *et al.*, 2011; Ray *et al.*, 2016; Southworth *et al.*, 2009; Tesson *et al.*, 2010). A drawback of the module-based DCA is that it leaves little room to control for the effect of expression level on the individual genes.

Validation also remains a challenge. While some studies report findings based on DCA (Hu *et al.*, 2009; Jiang *et al.*, 2016; Kostka and Spang, 2004; Mentzen *et al.*, 2009) to our knowledge they do not present any validation or replication of the differential coexpression. A strength of our work is that we use large quantities of data [as required to obtain robust coexpression results (Gillis and Pavlidis, 2011)], and we show that some ‘pure’ differential expression is reproduced in the GTEx RNA-seq data set. This was facilitated by the relative ease of finding data sets for tissues compared to more specialized conditions, but even so we were not able to find sufficient data to test more tissues. Similarly, it is unclear how sensitive previous studies have been. Thus most analyses for differential coexpression are likely to be underpowered or overwhelmed by noise, but also have an unclear false positive rate.

At this point we have yet to identify any clear biological significance for any of our pure links individually. This is partly because the nature of the genes makes it difficult to apply bioinformatic characterization methods to them. First, pure differential coexpression links are between genes expressed in all five tissues. This is in contrast to genes that are tissue-specific, which often have known, or more easily inferred, tissue-specific functions. Thus, we cannot ascribe a tissue-specific function of a pure link based on the expression pattern of the genes across tissues. Also, most of the genes expressed in all the tissues have at least one pure link—they are not a ‘special’ small subset of genes. Second, functional annotations of genes are rarely tissue-specific. That is, there are few cases where a gene expressed in multiple tissues has known distinct functions in those tissues (at least, such information is not captured by resources such as the gene ontology). Overall, this means there is a dearth of information on tissue-specific gene functions that are not attributable to differences in mean expression level, and thus evaluation of pure links is a challenge. Our results from a network-wide functional enrichment analysis showed that pure links in brain contribute to the tissue-specific enrichment of a few functional terms. This does not lead to any clear biological conclusion, but could assist the interpretation of results from other genomic or transcriptomic studies on the brain. Finally, unlike protein–protein interactions, coexpression is not a discrete property of a pair of genes nor an actual physical interaction. Coexpression is derived from the correlation matrix of the entire data set, so any tightly coexpressed pair of genes is essentially guaranteed to be part of a larger pattern (i.e. cluster or module) of genes. Therefore, coexpression links do not map cleanly to regulatory relationships nor protein–protein interactions. As a result, the core idea of DCA as reflecting ‘rewiring’ by necessity involves gene neighborhoods, modules or clusters. Taken together, these issues mean that the ability to extract specific (i.e. biochemical or physical) interactions from differential coexpression is severely limited. This limitation does not necessarily detract from the potential utility of tissue- or other context-specific data to improve the relevance of function predictions from coexpression.

## Funding

This research was supported by National Institute of Health [MH111099 to P.P., GM076990 to P.P.]; and Natural Sciences and Engineering Research Council of Canada [RGPIN-2016-05991].

*Conflict of Interest*: none declared.

## Supplementary Material

Supplementary Data
