# Supplementary material for: Differential coexpression in human tissues and the confounding effect of mean expression levels
Source: Bioinformatics. 2018 Jul 3;35(1):55–61. doi: 10.1093/bioinformatics/bty538 (PMC6298061; doi:10.1093/bioinformatics/bty538)
Supplement: Supplementary Data [file bty538_suppl_data.zip › SupplementaryText.pdf]

Supplementary Methods and Results

1. Dataset collection and QC

Affymetrix Microarray datasets:

We reanalyzed the data from raw CEL files using the RMA algorithm (Irizarry *et al.*, 2003). Outlier samples were detected and removed using the Gemma outlier detection algorithm (Zoubarev *et al.*, 2012). Batch effects were removed using the ComBat algorithm in the sva package in R (Leek *et al.*, 2012), with the batch information based on the date stamp in each CEL file. For mapping probesets to genes we used annotations from Gemma. For genes with multiple probesets the mean value was recorded as the expression level; however probesets with very low expression level (lowest tertile) were removed if the gene had at least one other probeset expressed at higher levels. The final data matrix included 18,494 genes and 3,563 samples (Supplementary Figure 1).

GTEx dataset:

The gene-level expression of GTEx version-6 was downloaded from the GTEx portal (Lonsdale *et al.*, 2013). We used data for blood, brain cortex, liver, lung and skeletal muscle. Genes which had read-counts > 0 in more than 75% of the samples were included in the analysis with no further normalization (Supplementary Table 9). In pairwise comparisons between Affymetrix and GTEx datasets, only genes which were marked as expressed in both datasets were considered.

Filtering of genes based on expression level for Affymetrix datasets

For each Affymetrix dataset, we filtered the genes based on their expression levels such that genes with mean expression below the first tertile were marked as not expressed, retaining 12,312 genes out

of the 18,494 genes on the platform. Genes which were expressed in > 80% of the datasets for a tissue were marked as expressed for that tissue (Supplementary Tables 6 to 10). For each tissue, only the expressed genes were considered for downstream analysis, including the measurements of network overlaps and densities. This was to avoid spurious correlations due to cross-hybridization (Ssupplementary Figure 2).

2. Gene ontology terms

The Gene Ontology (Ashburner *et al.*, 2000) data was downloaded on 31.03.2015. We used experimentally validated Gene Ontology terms under the Biological Process sub-group for human. Terms were filtered to have ≥ 10 and ≤ 200 genes assigned to them, resulting in 3,507 GO terms and 8,336 genes with one or more GO terms assigned to them.

3. Disease annotation for the genes

The Phenocarta (Tan *et al.*, 2016) data was extracted on 23.04.2018. The total count of annotations was 26,499, among 6,400 genes.

4. Tissue Specificity Score for GTEx data

To examine the reproducibility of our pure links as tissue-specific links in the GTEx datasets, we define TSS for a given pair of genes (g_i, g_j) and a tissue t from the five GTEx tissues: blood, brain-cortex, liver, lung and skeletal muscle the same as TSS for our TAN links. The only difference is that for GTEx we have just one coexpression value for each of the tissues. From total count of 506,221 tissue-specific links identified from TANs, we found that 263,302 (52%) have TSS > 0.56 in GTEx. The score 0.56 is the average of the TSS cut-off thresholds from the five tissues previously identified from Affymetrix datasets. From the total count of 80,637 pure links identified from our TANs, we found that 20,178 (25%) have TSS > 0.56 in GTEx. Table files in the supplementary data folder TSLinks_TSS have the GTEx

TSS for each of the tissue specific links, as well as the expression level of the paired genes in all the tissues.

5. Examining topological overlap for partners of pure links in the target tissue versus other tissues

We have identified 80,637 pure links in total across the five tissues (see Supplementary Table 8 for a break-down per tissue). For a given gene and network, we define the neighborhood of the gene in that network as the set of genes it is connected to. Based on the GBA principle, the neighbourhood of a gene in a coexpression network could suggest its functional role. Therefore, extreme changes in the neighborhood of a gene between the networks could suggest different functionality for the gene in those networks – and thus a different function for the gene in the tissues the networks are built from. Also, for a given pair of genes in a network, the similarity of their neighborhoods can be measured as their Topological overlap (TOP, (Yip and Horvath, 2007)). This similarity (high overlap) could also suggest functional similarity between the gene pair. We applied these ideas to examining if the presence of a pure link in a target TAN suggests higher TOP between the gene pair in that TAN, compared to the other TANs where the pure link is absent. For comparison and as a null, we also examined this for gene pairs with coexpression link present in multiple tissues: for a given pair of genes that have coexpression link in multiple TANs, how much does their TOP change between these TANs, and if the observed change is as extreme as what is observed for the gene pairs with pure link. The results from this part shows how much we can learn about a gene pair in the absence/presence of a pure differential coexpression link between them.

We used the Jaccard index for measuring the TOP. Each gene pair (g_i, g_j) which has a pure link in a target tissue gets five TOP measurements, called TOPJCCs, one from the target tissue TAN and four from the other tissue TANs. For simplicity, for each link we selected the maximum TOPJCC from the other

tissues for the comparisons. Supplementary Figure 5 shows the distribution of TOPJCC in the target tissues, compared to those from the maximum in the other tissues.

As explained, for the null we used gene pairs that have links in multiple TANs. We call these multi-tissue-links. We identified 368,411 gene pairs as such, which have coexpression links in two or more TANs. By definition, these gene pairs do not have tissue specific links and therefore the “target” and “other” tissues are not defined for them. To be able to compare their TOPJCC to those from the pure links, for each gene pair we marked the tissue with the highest TOPJCC the “target” and the tissue with the next highest TOPJCC as the “other” tissue. The plot in Supplementary Figure 5 shows that between the two genes that are expressed in multiple tissues, the presence of a pure link suggests higher TOPJCC in target tissue and lower TOPJCC in the other tissues (median 0.29 versus 0). This difference is not as extreme for the multi-tissue-links and is a feature unique to the pure link as a differential coexpression link. The difference in TOP overlap could suggest a change between functional similarity of the gene pairs in the target tissue compared to the other tissues for the pure links.

6. Contribution of the pure links to tissue-specific enrichment of functional terms in TSNs

For a given GO term *f* and a network N, we mark the term f_i as enriched in N if genes annotated with that term have significantly more links between them than the null, where null is the binomial distribution and the density of network N is the success probability. This was done for all our TSNs and TANs and for all the terms that had $\geq$20 genes annotated with them and also expressed in that tissue (Table file enrichedTerms_TAN_TSN_table.tsv). We hypothesized that for a given network, some of the functions would lose their enrichment significance if the pure links were removed, and this could further show which cellular processes are affected by the pure rewiring between the networks. We examined this for our TSNs in two steps: first we identified the functions which were exclusively enriched in each

of our TSNs. We found that 146, 91 and 43 functions are enriched exclusively in the brain, liver and lung TSNs respectively (the count of enriched functions for blood and skeletal muscle was too low for further analysis). Second, we removed the pure links and measured the significance of the enrichment for the same terms to identify the affected functional terms. When pure links were removed, the count of functions in brain dropped by 20, by 3 in liver and by 4 in lung. As a null comparison for each tissue, we removed sets of randomly selected tissue-specific links (the same count as the pure links) from each TSN, repeated 100 times. The count of drop-out functions for brain and lung (upon removal of the pure links) is less than that generated by removing random sets of links ($p < 0.05$). However, five functions in brain are specifically sensitive to the removal of pure links and not sensitive to the removal of random sets of links and expression-induced links (See table file brain_TSExclusvieFunctionTerms_linkRemoval-.txt, function IDs: 10821, 32388, 6650, 8654, 10506, Column: *significantCountIn100_random-PureLinkRemoval,* showing the count of times the function remained significant after removal of random sets of links). These functions only lost their significance in < 4 times out of 100 trials of random link removal.

7. *Contribution of expression-induced links to the tissue-specific enrichment of functional terms in TSNs*

In each TSN, links were marked as expression-induced if one or two of their genes were marked as exclusively expressed only in that tissue (see Supplementary Tables 11 and 12 for count of the genes and expression-induced links). The count of expression-induced links is much higher than the pure links in brain, liver and lung and therefore we hypothesized that they have larger contribution to the tissue-specific enrichment of functional terms than that of the pure links. We examined their contribution in two ways: 1. We compared the effect of removing expression-induced links to that from removal of the pure links, 2. We compared the removal of expression-induced links to a null.

For comparing removal of pure links to expression-induced links in brain, liver and lung, we selected random subsets of expression-induced links with the same count as the pure links and removed them from the network. We found that the count of drop-out functions upon the removal of subsets of expression-induced links is significantly higher than that of the pure links (100 trials, $p < 0.01$). The comparison to the null was similar to what we did for the pure links: we recorded the count of drop-out functions from the removal of the expression-induced links and compared it to the removal of random sets of links to the same count as the expression-induced links. When the expression-induced links were removed from the TSNs, the count of functions dropped by 67 in brain, 45 in lung and 27 in liver. Some of these functions were not affected by removal of random sets of links – their tissue-specific enrichment was primarily due to expression-induced links (see table files [tissue]_TSNExclusiveFunctionTerms_linkRemoval.txt, column: significantCountIn100_randomEILinkRemoval, showing count of times the function remained significant after removal of random sets of links).

8. Enrichment of the Disease Ontology terms in the networks

We also examined the enrichment of the disease terms in the networks similar to the functional terms. We had 18 and 30 terms exclusively enriched in brain and lung TSNs (the count of terms enriched in other tissues was too small for further analysis and interpretation). We found that only 1 term in brain and 5 in liver are sensitive to the removal of pure links, but the same terms were also sensitive to the removal of random sets of links (see table files [tissue]_TSExclusiveDiseaseTerms_linkRemoval.txt)

9. Descriptions of supplementary files

The file enrichedTerms_TAN_TSN_table.tsv has the list of functional terms enriched in TANs and TSNs. There are also 15 table files in three groups. Each group has five files, one for each tissue, identified with the tissue name in the file name.

TSlinksTSS_[tissue]_pureLinks_GTEx_exp.csv (5 files, one for each tissue)

These files have 18 columns and each row represents a TSN links, for which its gene symbols, correlation bins in GTEx and the average log2 expression values of the genes are represented.

Column	Header	Description
1-2	gene_A, gene_B	Gene symbols
3-7	[tissue]_corr	Correlation bin for the gene pair from GTEx dataset
8	GTEx_TSS	TSS from the GTEx, calculated as explained in Supplementary Material section 4
9-18	gene_[A or B][tissue]	The average log2 expression level for the genes in each of the tissues

[tissue]_TSExclusiveFunctionTerms_linkRemoval.txt

These files have 15 columns, each row represent a functional term in GO.

Column	Header	Description
1	functionID	GO ID of the functional term
2	functionName	Name of the function in GO
3	geneCount	Count of genes associated with that term in the TSN of the tissue
4	linkCount(lc)	Count of links between the genes associated with that term in the TSN of the tissue
5	lc_minusPureLinks	This is linkCount(lc) minus pure links from the network
6	lc_minusExpressionInducedLinks	This is linkCount(lc) minus expression-induced links from the network

7	1-ratioOfPureLinks	Ratio of non-pure linkCount(lc)
8	1-RatioOfExpressionInducedLinks	Ratio of non-expression-induced linkCount(lc)
9	SigAfterPureRemoval	If the term is still significant after removing of the pure links
10	SigAfterExpressionInducedRemoval	If the term is still significant after removing of the expression-induced links
11	log10Pvalue_plus1-e20	The p-value for the significance of function
12	log10PvalueAfterPureRemoval_plus1-e20	The p-value for the significance of function after removing the pure links from the network
13	log10PvalueAfterEIremoval_plus1-e20	The p-value for the significance of function after removing the expression-induced links
14	significantCountIn100_randomPureLinkRemoval	Count of times the function was significant in 100 trials of removing random sets of links to the count of pure links
15 or 16	significantCountIn100_randomEILinkRemoval_pureCount	Count of times the function was significant in 100 trials of removing expression-induced links to the count of pure links (this was done only for brain, liver and lung)
15 or 16	significantCountIn100_randomEILinkRemoval	Count of times the function was significant

		in 100 trials of removing random sets of links to the count of expression-induced links

[tissue]_TSExclusiveDiseaseTerms_linkRemoval.txt

These files have 15 columns for blood and skeletal musche and 16 columns for brain, liver and lung.

Each row represent a diseaseal term in GO.

**Column**	**Header**	**Description**
1	DOID	Disease-ontology ID of the disease term
2	Name	Name of the disease in disease ontology
3	geneCount	Count of genes associated with that term in the TSN of the tissue
4	linkCount(lc)	Count of links between the genes associated with that term in the TSN of the tissue
5	lc_minusPureLinks	This is linkCount(lc) minus pure links from the network
6	lc_minusExpressionInducedLinks	This is linkCount(lc) minus expression-induced links from the network
7	1-ratioOfPureLinks	Ratio of non-pure linkCount(lc)
8	1-RatioOfExpressionInducedLinks	Ratio of non-expression-induced linkCount(lc)
9	SigAfterPureRemoval	If the term is still significant after removing of the pure links
10	SigAfterExpressionInducedRemoval	If the term is still significant after removing of the expression-induced links
11	log10Pvalue_plus1-e20	The p-value for the significance of disease
12	log10PvalueAfterPureRemoval_plus1-e20	The p-value for the significance of disease after removing the pure links from the network

13	log10PvalueAfterEIremoval_plus1-e20	The p-value for the significance of disease after removing the expression-induced links
14	significantCountIn100_-randomPureLinkRemoval	Count of times the disease was significant in 100 trials of removing random sets of links to the count of pure links
15 or 16	significantCountIn100_-randomEILinkRemoval_pureCount	Count of times the disease was significant in 100 trials of removing expression-induced links to the count of pure links (this was done only for brain, liver and lung)
15 or 16	significantCountIn100_-randomEILinkRemoval	Count of times the disease was significant in 100 trials of removing random sets of links to the count of expression-induced links

10. Supplementary figures

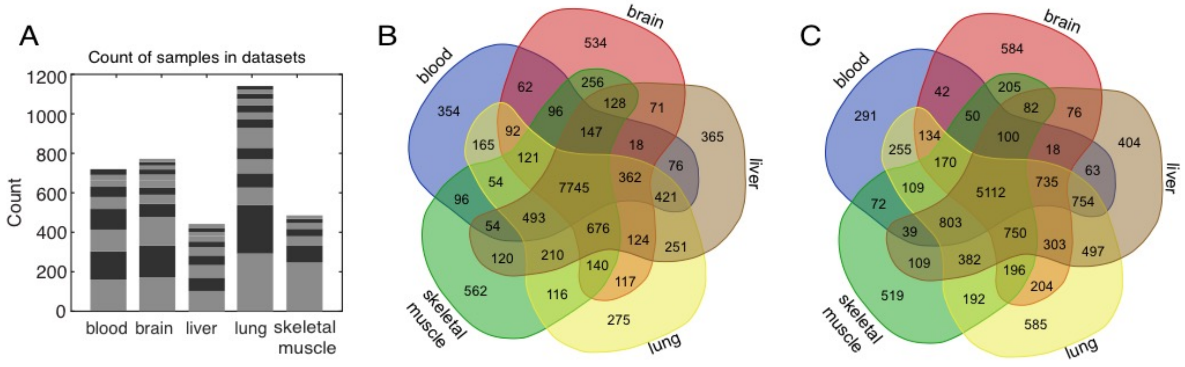

Supplementary Figure 1

(A) Distribution of the count of samples for different datasets in each of the tissues. The lighter and darker colors mark the count of samples for different datasets. (B) Overlap of the genes marked as

expressed in the five tissues. (C) Overlap of the genes with any edges in the relevant TAN networks. Venn diagram built in http://bioinformatics.psb.ugent.be/webtools/Venn/

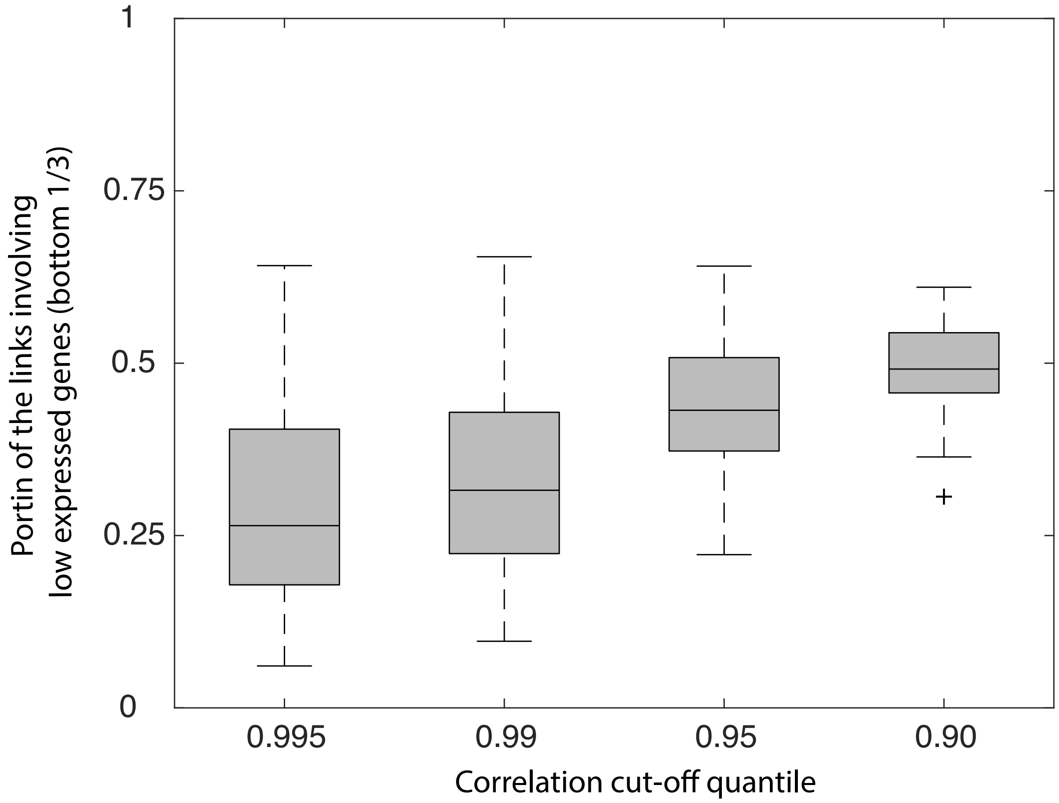

Supplementary Figure 2

Proportion of coexpression links having one or two of their genes marked as "not expressed" in the datasets. Each box plot represents the portion of the links for each of the 53 datasets at a correlation cut-off threshold.

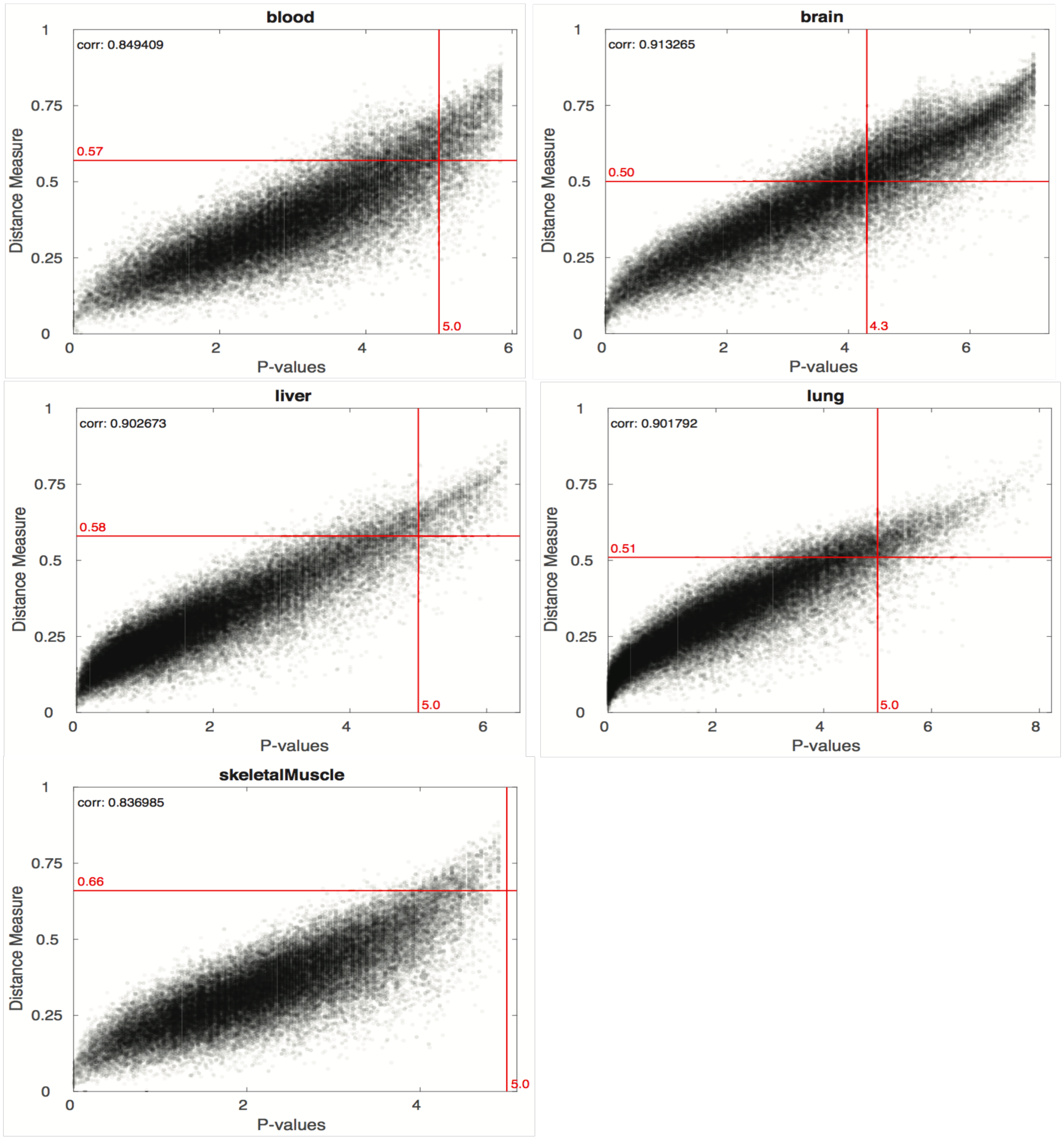

Supplementary Figure 3

Comparison of the TSS and p-values from Wilcoxon-rank-sum test. Scatter plots are the unadjusted $-\log_{10}$(p-values) of the Wilcoxon Rank Sum test and the Tissue Specificity Scores. Red lines are at the cut-off threshold for FDR < 0.01. Results from the two methods are highly correlated.

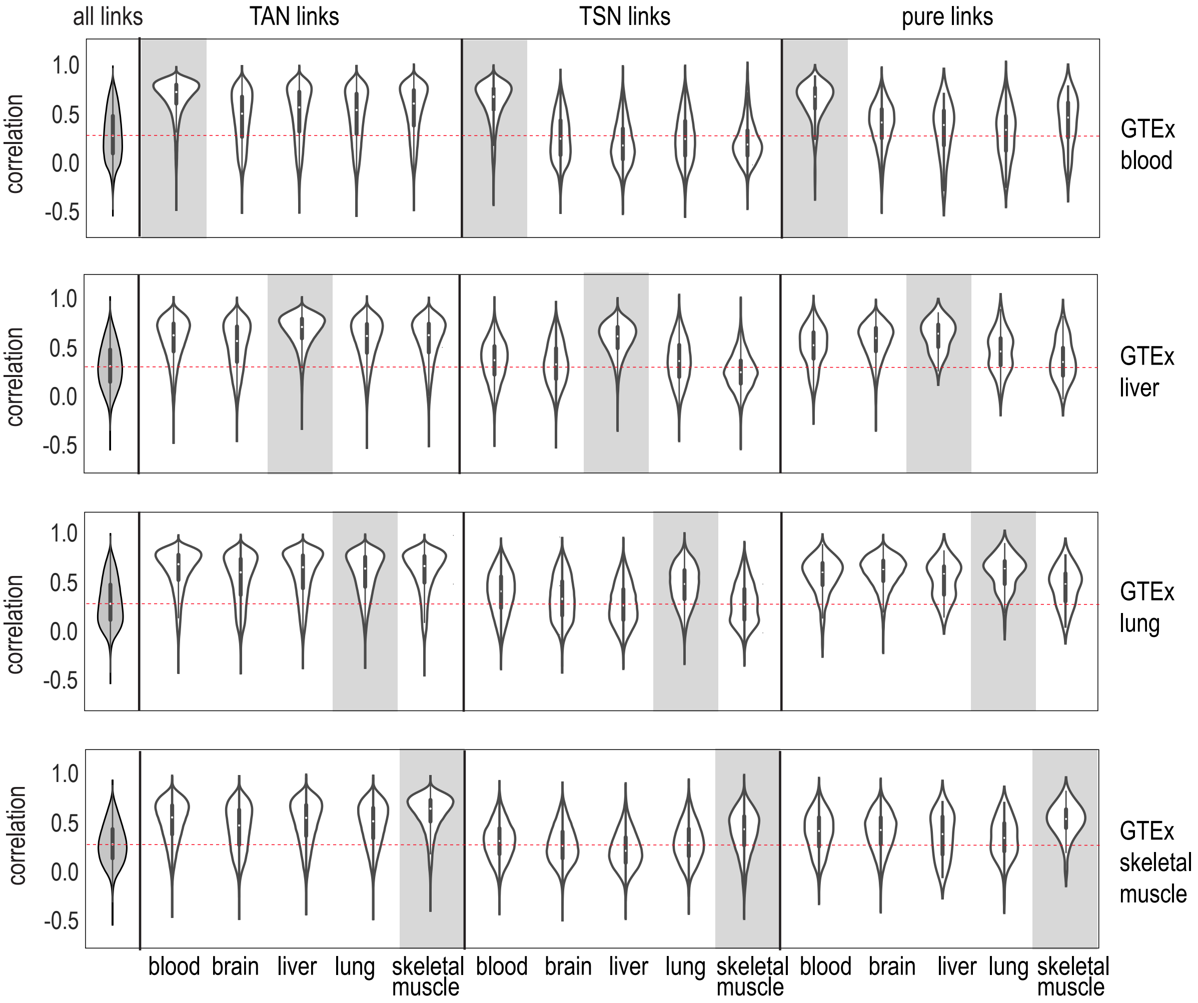

Supplementary Figure 4

Reproducibility of the TAN, TSN and pure links for GTEx datasets blood, liver, lung and skeletal muscle (plot for GTEx brain-cortex is in Figure-3).

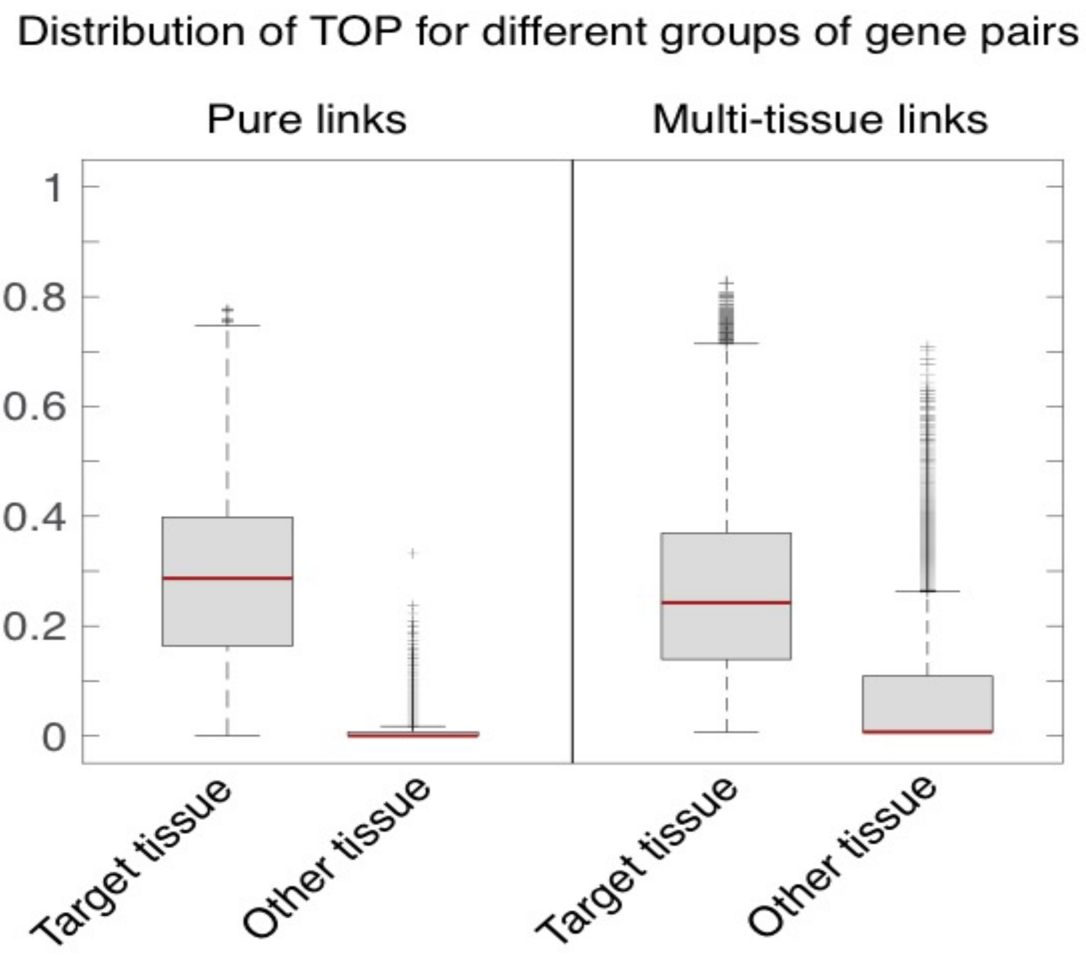

Supplementary Figure 5

Distribution of topological overlap for different groups of gene pairs that are expressed in multiple tissues. Left: distribution of TOP for partners of pure links in the target tissue, versus the maximum TOP from the other tissues. Right: distribution of TOP for gene pairs with coexpression links in multiple tissues. The tissue with highest TOP was considered as “target” and the maximum TOP after that was considered as the “other” tissue. While for the pure differential coexpression links the TOP from “other tissue” is close to zero for almost all the links, for a multi-tissue coexpression link the TOP from the other tissue is above zero for more than half of the links.

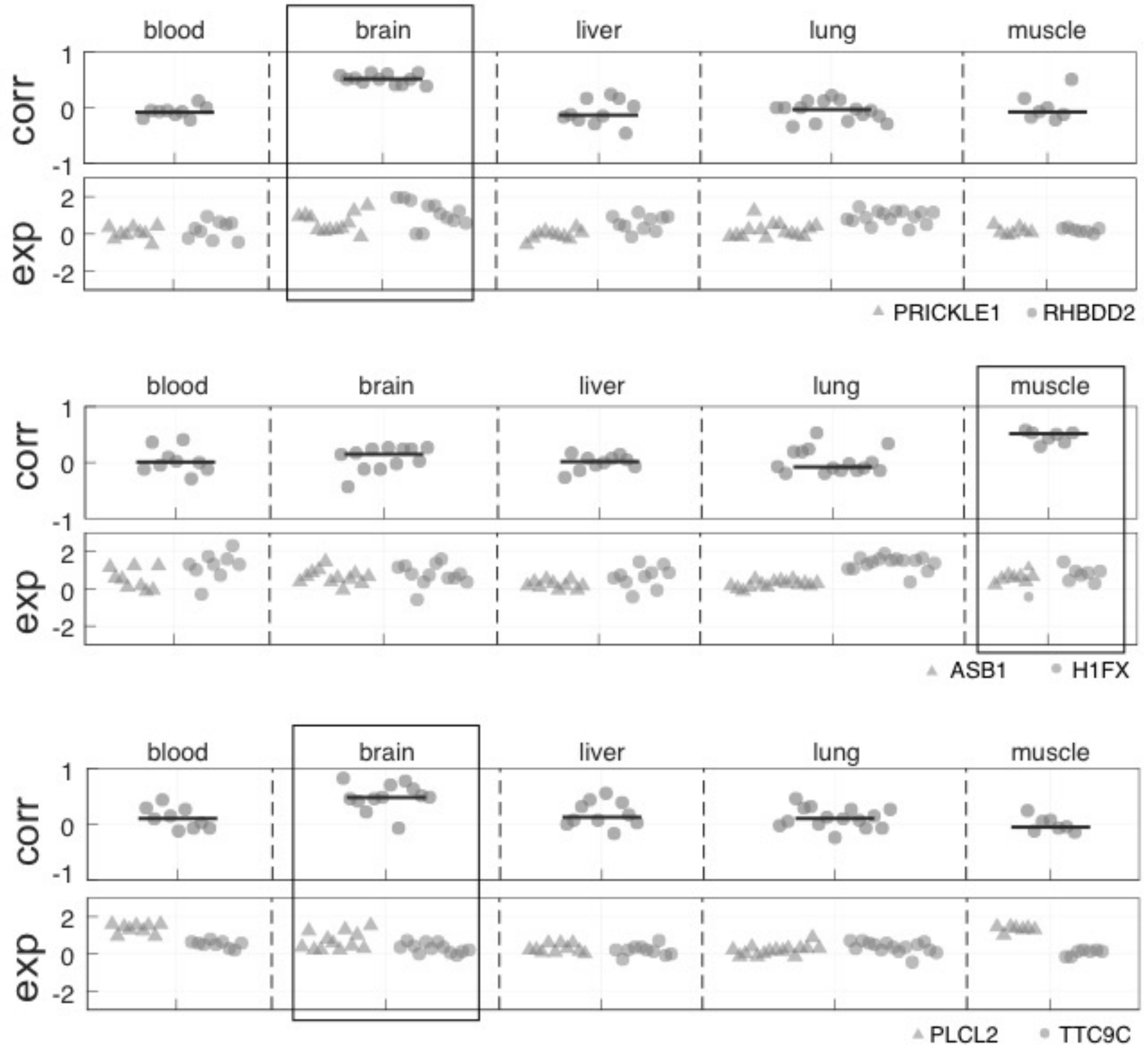

Supplementary Figure 6

Additional examples of pure links (randomly selected). As in Figure 1 in the main text, for each link, Pearson correlation values and normalized gene expression values for each of the datasets is plotted. The target tissue is marked with the black box.

Supplementary Tables

Supplementary Table 1: Affymetrix blood datasets

GEO ID	#Samples used/ #Initial Samples	#Samples removed in batch effect/ as outlier	Notes
GSE13849	120/110	10/0	-
GSE16028	105/109	1/3	-
GSE19314	60/65	0/5	-
GSE25507	143/145	1/1	-
GSE26050	29/33	1/3	-
GSE27263	52/92	0/0	40 samples from nasal biopsy removed
GSE27562	159/162	0/3	-
GSE33223	29/30	0/1	-
GSE7753	31/31	0/0	-

Supplementary Table 2: Affymetrix brain datasets

GEO ID	#Samples used/ #Initial Samples	#Samples removed in batch effect/ as outlier	Notes
GSE11882	172/173	0/1	-
GSE13564	37/44	2/5	-
GSE17612	45/51	0/6	-
GSE20146	16/20	0/4	-
GSE28160	31/38	7/0	-
GSE3526	145/353	0/2	Samples from different tissues
GSE35864	68/72	0/4	-
GSE4036	26/28	0/2	-
GSE4757	20/20	0/0	-

GSE5281	160/161	1/0	-
GSE7621	18/25	7/0	-
GSE9770	34/34	0/0	-

Supplementary Table 3: Affymetrix liver datasets

GEO ID	#Samples used/ #Initial Samples	#Samples removed in batch effect/ as outlier	Notes
GSE12720	63/63	0/0	-
GSE14668	27/28	0/1	-
GSE15235	43/47	2/2	-
GSE17183	104/108	2/2	-
GSE19665	20/20	0/0	-
GSE26622	20/24	0/4	-
GSE28619	21/22	0/1	-
GSE38663	49/52	0/3	-
GSE40873	28/49	?/3	The rest of samples seem to have dropped at batch effect
GSE6764	68/75	3/4	

Supplementary Table 4: Affymetrix lung datasets

GEO ID	#Samples used/ #Initial Samples	#Samples removed in batch effect/ as outlier	Notes
GSE10245	53/58	0/3	-
GSE10445	70/72	2/0	-
GSE11729	59/60	0/1	-
GSE12667	73/75	0/2	-
GSE14334	36/38	0/2	-

GSE18842-1	43/45	0/2	The normal samples from GSE18842
GSE18842-2	45/46	0/1	The tumor samples from GSE18842
GSE21369	25/29	0/4	-
GSE27262-1	19/25	3/3	The healthy samples from GSE27262
GSE27262-2	21/25	3/1	The tumor samples from GSE27262
GSE27716	34/40	6/0	-
GSE28571	89/100	1/11	-
GSE30219	293/307	6/0	The 8 normal samples were removed
GSE31210	245/246	0/1	-
GSE37768	35/37	1/2	-

Supplementary Table 5: Affymetrix skeletal muscle datasets

GEO ID	#Samples used/ #Initial Samples	#Samples removed in batch effect/ as outlier	Notes
GSE13070	247/364	?/5	Samples from adipose tissue removed
GSE17674	17/62	1/0	44 tumor samples were removed
GSE19420	38/42	0/4	-
GSE25462	48/50	0/2	-
GSE35659	22/24	1/2	-
GSE47881	85/89	0/4	-
GSE33223	29/30	0/1	-
GSE7014	29/36	1/0	6 normal samples were removed

*Supplementary Table 6: TAN attributes *ND = Node Degree*

attribute	blood	brain	liver	lung	skeletal muscle
#expressed genes	10,356	10,689	11,261	11,362	11,014
#genes with ND > 0	8,747	8,761	10,227	11,181	8,890
#links	380,875	1,118,791	496,476	752,298	358,029
Density	0.007	0.020	0.008	0.012	0.006

*Supplementary Table 7: TSN attributes *ND = Node Degree*

attribute	blood	brain	liver	lung	skeletal muscle
#genes with ND > 0	5,948	6,422	4,320	8,140	3,712
#links	33,594	358,531	41,706	62,209	10,181
Density	4e-4	4e-3	1e-3	5e-4	4e-4

*Supplementary Table 8: Pure attributes * ND = Node Degree*

attribute	blood	brain	liver	lung	skeletal muscle
#genes with ND > 0	3,468	3,876	1,452	3,521	998
#links	8,476	62,179	2,440	6,620	922
Density	1.6e-4	1.1e-3	3.8e-5	1e-4	1.5e-5

*Supplementary Table 9: GTEx binary networks attributes *ND = Node Degree*

attribute	blood	brain_cortex	liver	lung	skeletal muscle
#expressed Genes	14,089	15,996	15,213	15,882	14,810
#Genes expressed in Affymetrix dataset	9,978	10,389	10,910	11,129	10,301
#genes with ND > 0	6,989	7,548	8,100	6,434	7,996
#links	365,775	1,095,477	484,903	735,392	336,358
Density	0.007	0.020	0.008	0.012	0.006

Supplementary Table 10: Count of tissue-specific genes, FDR < 0.1, log transformed FC = 0

blood	brain	liver	lung	skeletal muscle
254	129	250	114	212

Supplementary Table 11: Count of genes exclusively expressed in each of the tissues

blood	brain	liver	lung	skeletal muscle
354	543	365	275	562

Supplementary Table 12: Count of expression-induced links

blood	brain	liver	lung	skeletal muscle
4,714	125,057	16,787	11,953	4,237

References

Ashburner,M. *et al.* (2000) Gene ontology: tool for the unification of biology. The Gene Ontology Consortium. *Nat. Genet.*, **25**, 25–29.

Irizarry,R.A. *et al.* (2003) Exploration, normalization, and summaries of high density oligonucleotide array probe level data. *Biostatistics*, **4**, 249–264.

Leek,J.T. *et al.* (2012) The sva package for removing batch effects and other unwanted variation in high-throughput experiments. *Bioinforma. Oxf. Engl.*, **28**, 882–883.

Lonsdale,J. *et al.* (2013) The Genotype-Tissue Expression (GTEx) project. *Nat. Genet.*, **45**, 580–585.

Tan,P.P.C. *et al.* (2016) Interactive Exploration, Analysis and Visualization of Complex Phenome-Genome Datasets with ASPIREdb. *Hum. Mutat.*, n/a-n/a.

Yip,A.M. and Horvath,S. (2007) Gene network interconnectedness and the generalized topological overlap measure. *BMC Bioinformatics*, **8**, 22.

Zoubarev,A. *et al.* (2012) Gemma: a resource for the reuse, sharing and meta-analysis of expression profiling data. *Bioinforma. Oxf. Engl.*, **28**, 2272–2273.
